# Plasma Exchange for Thrombotic Microangiopathy Associated With Rectal Cancer Presenting With Acute Kidney Injury: A Case Report

**DOI:** 10.7759/cureus.109120

**Published:** 2026-05-18

**Authors:** Yuanye Li, Weixiu Wang, Manyu Zhang, Chaoyuan Li, Dingwei Yang

**Affiliations:** 1 Department of Nephrology, Tianjin Hospital, Tianjin University, Tianjin, CHN

**Keywords:** acute kidney injury, kidney biopsy, plasma exchange, rectal cancer, thrombotic microangiopathy

## Abstract

Thrombotic microangiopathy (TMA) is an acute syndrome characterized by microangiopathic hemolytic anemia, thrombocytopenia, and ischemic end-organ damage resulting from platelet-rich microthrombi obstructing the microvasculature. The kidney is particularly vulnerable to TMA-mediated injury due to its numerous microvessels, often leading to acute kidney injury (AKI). AKI induced by TMA can manifest in oncological conditions, although infrequently. Rectal cancer presenting with TMA-related AKI as the initial manifestation is even rarer, posing a diagnostic challenge for nephrologists. We describe a 62-year-old man admitted with AKI followed by severe thrombocytopenia and anemia. During the diagnostic workup to exclude thrombotic thrombocytopenic purpura, the patient achieved complete hematological recovery after hemodialysis and plasma exchange (PEX), which enabled the performance of a renal biopsy. The underlying etiology was TMA on renal biopsy. However, persistent elevation of lactate dehydrogenase and new‑onset of bone pain prompted further evaluation. The magnetic resonance imaging scan and positron emission tomography-computed tomography subsequently confirmed rectal cancer with bone and liver metastases. This case illustrates that AKI secondary to cancer-related TMA may present as the initial clinical manifestation of an underlying malignancy. Renal biopsy plays a crucial role in establishing a definitive diagnosis and guiding subsequent management. Furthermore, PEX was associated with hematological improvement in this patient, but its overall clinical benefit remains to be determined as the patient remained dialysis‑dependent.

## Introduction

Thrombotic microangiopathy (TMA) covers a broad range of disorders characterized by microangiopathic hemolytic anemia, thrombocytopenia, and end-organ damage, including thrombotic thrombocytopenic purpura (TTP), hemolytic uremic syndrome (HUS), and secondary forms associated with conditions such as malignancy, drugs, infection, autoimmune disease, and malignant hypertension.

In patients with cancer, TMA is uncommon and may be associated with either the underlying cancer or its treatment, such as chemotherapy. Cancer‑related TMA is directly driven by the underlying malignancy (often metastatic adenocarcinoma) and requires prompt anti‑cancer therapy, whereas chemotherapy‑induced TMA results from dose‑dependent or drug toxicity and typically responds to drug discontinuation and supportive care. The prognosis of cancer‑related TMA is generally poor and linked to tumor progression, while many chemotherapy‑induced TMA cases, particularly those caused by anti-vascular endothelial growth factor (anti-VEGF) agents, can show partial or complete recovery after withdrawal of the drug [1]. Lung, gastric, prostate, and breast cancers are the most frequently implicated solid cancers, while TMA reported in rectal cancer is rare [2]. To our knowledge, literature on rectal cancer directly triggering TMA is extremely limited, with most reported cases in colorectal cancer describing TMA induced by chemotherapy or targeted agents rather than by the tumor itself [3]. Furthermore, management of previously reported gastrointestinal malignancy‑related TMA cases remains challenging, and prognosis is uniformly poor, underscoring the urgent need for prompt diagnosis and treatment [4]. Occasionally, TMA may be a characteristic manifestation of occult malignant tumors. Approximately 3% of patients initially diagnosed with TTP ultimately have an underlying malignancy [5]. Renal microvessels are particularly susceptible to TMA-mediated damage. The formation of microthrombi in arterioles and capillaries can impair renal blood flow and filtration function, usually manifesting as acute kidney injury (AKI) and accompanied by hematuria and proteinuria, making it the most prominent extra-hematological manifestation.

The diagnostic criteria for TTP are the severe deficiency of a disintegrin and metalloproteinase with a thrombospondin type 1 motif, member 13 (ADAMTS13) activity (<10%), which may be due to gene mutations or autoantibody-mediated inhibition. ADAMTS13 is a von Willebrand factor (vWF)-cleaving protease that limits platelet adhesion and aggregation at sites of endothelial injury. Normal or mildly reduced activity of ADAMTS13 points toward secondary causes such as HUS, cancer‑related TMA, or drug‑induced TMA [6]. Plasma exchange (PEX) is the standard treatment for TTP and should be initiated urgently, as it can rapidly remove inhibitory anti-ADAMTS13 autoantibodies and vWF multimers while supplementing functional ADAMTS13 to prevent microvascular thrombosis and irreversible end-organ damage. Unlike primary TTP, which is highly responsive to PEX, cancer‑related TMA generally carries a poor prognosis, with reported 30‑day mortality rates approaching 50%, largely due to the advanced stage of the underlying malignancy [7]. Given the time required to obtain ADAMTS13 results, and considering the rapid progression and poor prognosis of TTP, prompt initiation of PEX is recommended while awaiting the results [8]. Despite its poor prognosis, cancer‑related TMA remains underrecognized, and there is no consensus on optimal management. This case report aims to contribute valuable clinical data to this ongoing discussion. We report a case of AKI secondary to TMA associated with rectal cancer, as confirmed by renal biopsy. While awaiting TTP test results, the patient received two daily PEX sessions and achieved complete hematological recovery.

## Case presentation

A 62-year-old man presented with a two-day history of dyspnea, oliguria, and lower limb edema. There were no neurological symptoms. He reported normal renal function and urinalysis before admission. Notably, the patient reported no weight loss, changes in bowel habits, or abdominal pain before admission. The symptom of fatigue was gradually worsening over the preceding two weeks, which he attributed to aging. His urine output remained 100-200 mL per 24 hours, and blood pressure was elevated to 192/98 mmHg. On admission, his serum creatinine was elevated at 891 µmol/L. Serum albumin was 35.8 g/L, and lactate dehydrogenase was 321 U/L. Urinalysis revealed mild proteinuria and microscopic hematuria (10 red blood cells per high-power field, 60% of which were dysmorphic). Test results for myocardial enzymes, autoimmune diseases, infectious diseases, and tumor markers were unremarkable. Abdominal ultrasonography demonstrated normally sized kidneys. A computed tomography (CT) scan of the chest revealed pneumonia with pleural and pericardial effusions. His platelet count decreased to 29 × 10⁹/L, and hemoglobin decreased to 92 g/L. Due to the suspected diagnosis of TTP, PEX was initiated on the same day that the patient developed severe thrombocytopenia, pending investigations for the underlying cause. Hemodialysis every other day was initiated due to oliguria. Methylprednisolone (20 mg daily) and intravenous human immunoglobulin were also commenced.

Approximately one week after the initiation of PEX, further laboratory results became available, which showed a slightly elevated reticulocyte count (120.2 × 10⁹/L; reference range, 24-84 × 10⁹/L) and percentage (3.62%; reference range, 0.5%-1.5%). The vWF level was 373.0% (reference range, 50%-160%), and ADAMTS13 activity was 134.06% (reference range, 42.16%-126.37%), effectively excluding TTP. Tests for anti-ADAMTS13 IgG autoantibody, the Coombs test, and anti-platelet factor 4/heparin antibodies were all negative. Blood and urine cultures showed no growth. Serum and urine electrophoresis and immunofixation were negative for monoclonal gammopathy. Complement C3 and C4 levels were normal, and the absence of precursor infection made HUS unlikely. There was no recent exposure to drugs known to cause TMA. After the results were reported, two sessions of daily PEX were conducted, but were not continued. However, after receiving existing treatment, the patient's platelet count increased to 113 × 10 ⁹/L and hemoglobin level increased to 100 g/L in five days (Table 1).

**Table 1:** Change in laboratory results before and after plasma exchange. PEX, plasma exchange; ALT, alanine transaminase; AST, aspartate aminotransferase; LDH, lactate dehydrogenase

Biochemical analysis	Before the PEX	Five days after the last session of PEX	Reference interval
				Hemoglobin (g/L)	92	100	130-175
White cell count (× 10⁹/L)	11.6	9.3	3.5-9.5				
				Platelet (× 10⁹/L)	29	113	125-350
Reticulocyte count (%)	3.6	-	0.5-1.5				
				Reticulocyte count (× 10⁹/L)	120.2	-	24-84
Serum potassium (mmol/L)	3.6	4.2	3.5-5.3				
				Serum creatinine (µmol/L)	891	747	57-111
Serum albumin (g/L)	35.8	40.5	40-55
				Serum bilirubin (µmol/L)	8.6	13.9	3.4-17.1
Serum ALT (U/L)	18.8	23.9	9-50				
				Serum AST (U/L)	20.7	37.1	15-40
Serum LDH (U/L)	321	409	120-250				
				D-Dimer (ng/mL)	2100	1900	0-300

A renal biopsy was performed approximately two weeks after admission. Light microscopy of the renal biopsy specimen revealed 14 glomeruli, one of which showed ischemic global sclerosis. Segmental double‑contour formation (glomerular basement membrane duplication) was observed in some areas. Mild thickening of the arteriolar walls was noted. Immunofluorescence microscopy showed no evidence of immune complex deposition. Electron microscopy examined two glomeruli, demonstrating marked vacuolar degeneration of some capillary endothelial cells, segmental expansion of the subendothelial space (lamina rara interna) within capillary loops, and occasional red blood cells within capillary lumina. No definite electron‑dense deposits were identified (Figure 1). These ultrastructural findings confirmed the diagnosis of secondary TMA. 

**Figure 1:**
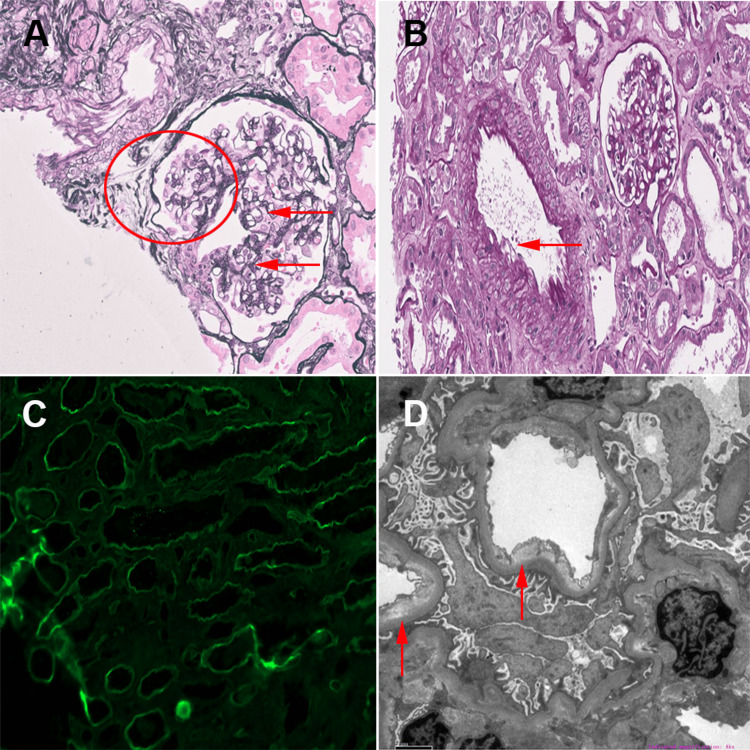
Pathological findings of renal biopsy. (A) The circles and arrows indicate the formation of segmental *double contour* structures of the glomerular basement membrane (PASM staining ×400). (B) Mild arteriolar proliferation and hypertrophy are indicated by an arrow (PAS staining ×200). (C) Immunofluorescent staining showed no deposition of immune complexes. (D) Swollen glomerular endothelial cells and expansion of the lamina rara interna are shown by arrows (electron microscopy ×4,000). PASM, Periodic Acid–Silver Methenamine

Approximately two weeks after the biopsy, the patient reported low back pain. The magnetic resonance imaging scan for the lumbar showed pathological fractures due to metastasis (Figure 2). Approximately one week later, positron emission tomography (PET)‑CT revealed the patient's rectal cancer, accompanied by bone and liver metastases. After one month of anti‑tumor therapy initiated by the oncology department, the patient’s urine output increased slightly from approximately 100-150 mL/24 hours to 400-500 mL/24 hours. Nevertheless, he remained dialysis‑dependent.

**Figure 2:**
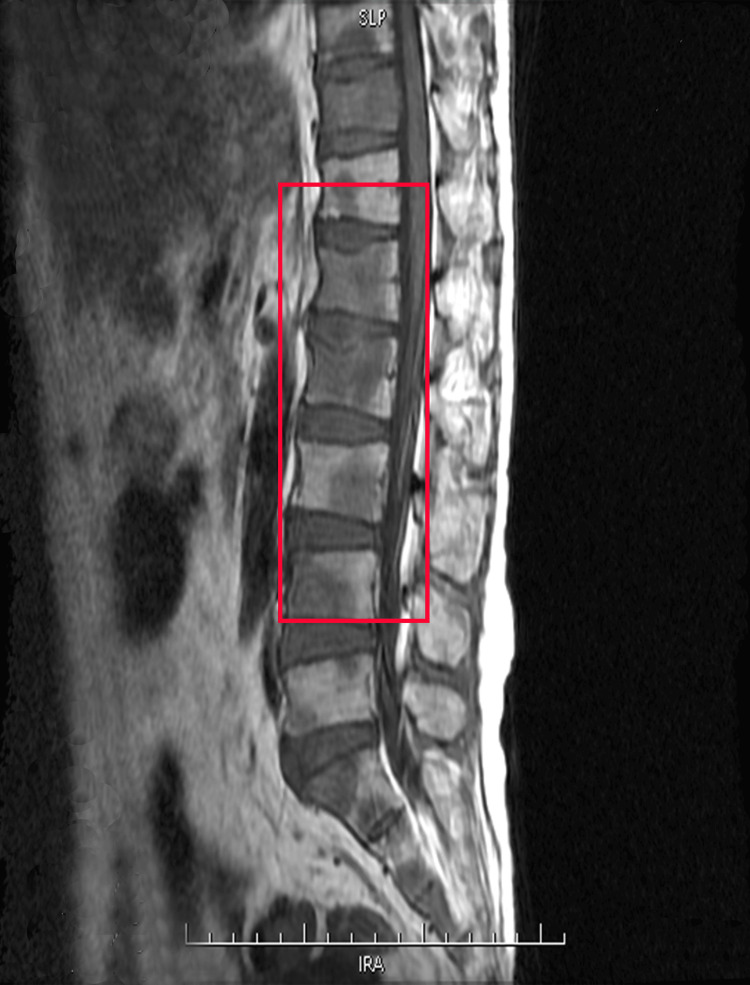
Magnetic resonance imaging scan of the lumbar spine. The red box shows pathological fractures of the T11, L1, and L2 vertebrae, suspected to be malignant lesions.

## Discussion

Cancer can directly trigger TMA, sometimes as the first warning sign, sometimes in advanced disease. Several mechanisms have been proposed to explain the pathogenesis of cancer-related TMA. First, direct activation of the coagulation cascade by tumor cells - particularly in mucin-secreting adenocarcinomas - can lead to excessive thrombin generation, platelet consumption, and fibrin deposition in the microvasculature, ultimately triggering TMA [9]. Second, dysregulation of the alternative complement pathway has been implicated, either as a paraneoplastic phenomenon or secondary to specific chemotherapeutic agents such as gemcitabine and oxaliplatin, with complement activation contributing to endothelial injury and microthrombus formation [10]. Third, tumor microemboli can cause direct mechanical damage to the microvascular endothelium, leading to intimal proliferation, fibrinoid necrosis, and subsequent TMA [9]. Fourth, severe deficiency of ADAMTS13 activity (<10%), although classically associated with TTP, has been observed in some cases of cancer-related TMA, potentially due to autoantibody formation against the metalloprotease-1 [10]. Fifth, the pro-inflammatory milieu induced by cytokines secreted by tumor cells, such as interleukin-6 and tumor necrosis factor-alpha, can promote endothelial activation, platelet hyperaggregability, and a prothrombotic state, further contributing to TMA pathogenesis [1,9].

The renal pathological manifestations of TMA not only vary depending on the primary disease, but also evolve with the progression of the condition. In the acute phase, glomerular or small artery thrombosis is the characteristic change, and the presence of arterial intimal edema (mucus/mucinous changes) or fibrin also has diagnostic significance. However, it is easy to overlook in limited samples. Endothelial cell proliferation (especially in pregnancy-related syndromes) and mesangial lysis are typical manifestations in the glomeruli. In the chronic phase, there may be diffuse irregular thickening of the glomerular basement membrane, segmental *double contour *changes, and arterial onion-like changes caused by intimal hyperplasia. With repeated circulation of the acute and chronic stages of the disease, both acute and chronic lesions can be observed simultaneously in a single sample [11,12]. The renal pathology of this patient showed typical TMA manifestations, including endothelial swelling and segmental *double contour* structures of the glomerulus. The results of renal biopsy pathology combined with ADAMTS13 activity helped us clarify the diagnosis of TMA and exclude TTP. This guided us in determining the next steps of examination and treatment plan. When AKI is a significant symptom and the cause is difficult to identify, renal biopsy should be performed as thoroughly as possible, and pathological features should be carefully distinguished to avoid a missed diagnosis [11].

Notably, the patient’s serum lactate dehydrogenase (LDH) increased slightly from 321 to 409 U/L after PEX. While a decrease in LDH is typically expected in TTP patients responding to PEX, it is crucial to interpret this finding within the overall clinical picture. His platelet count normalized, and hemoglobin improved, indicating a successful hematological response to PEX. Furthermore, the diagnosis of TTP was unequivocally excluded by a normal ADAMTS13 activity of 134.06%. Therefore, the LDH elevation cannot be attributed to persistent TMA. Instead, it is far more likely to reflect the progression of the underlying malignancy. This interpretation is strongly supported by the subsequent PET-CT findings of extensive bone and liver metastases. Elevated serum LDH is a well-established biomarker of poor prognosis and disease progression in metastatic solid tumors, particularly in colorectal cancer, where it correlates with tumor burden, metabolic activity, and worse survival outcomes [13]. In this context, the rising LDH during the patient’s hospitalization is most consistent with disease progression rather than treatment failure. This case highlights that in cancer patients with TMA, once TTP is confidently excluded, LDH should be interpreted with caution and its value as a marker of tumor progression should be prioritized.

The treatment of cancer-related TMA involves promptly removing the cancer, actively administering anticancer treatment, providing supportive care, and discontinuing medications that may exacerbate disease progression [14]. PEX is a controversial treatment for cancer related TMA. Multiple studies suggest that routine PEX should not be performed in cancer related TMA due to its unconfirmed effectiveness, increased risks of central venous catheter related complications, venous thrombotic events, and plasma reactions [12,15,16]. However, our patient showed significant clinical improvement and initial hematological remission after receiving two sessions of PEX treatments for suspected TTP at the beginning. The effectiveness of PEX has also been reported in cases similar to ours, including gastric signet ring cell carcinoma and metastatic pancreatic carcinoma [17,18]. A critical comparison with previously reported cases reveals both similarities and important differences. In a case of metastatic pancreatic adenocarcinoma, Mok and Cheung [17] reported complete hematological and renal recovery after four PEX sessions, whereas in a case of gastric signet‑ring cell carcinoma, Candar et al. [18] observed significant hematological improvement after 14 PEX sessions without renal involvement. Our patient achieved hematological normalization after only two PEX sessions, fewer than both reported cases, but remained dialysis‑dependent, likely due to more established chronic TMA changes on renal biopsy. On histopathological examination, the patient exhibited glomerular basement membrane duplication (double contour), arteriolar intimal proliferation and hypertrophy, and endothelial swelling. These findings are the hallmark of chronic TMA, reflecting long-standing endothelial injury. The presence of established chronic architectural changes, particularly when combined with tubulointerstitial fibrosis, indicates that the injury has progressed beyond the point of possible complete structural reversal [19]. Reported renal outcomes in cancer‑related TMA are variable, but persistent dialysis dependence is common, especially when chronic TMA changes are present on biopsy. Our patient’s chronic histologic findings likely explain his lack of renal recovery despite hematologic improvement.

These comparisons suggest that the hematological response to PEX in cancer‑related TMA can be rapid and does not require a fixed number of sessions, but renal recovery depends on the extent and chronicity of pre‑existing microvascular injury. The optimal duration of PEX and its impact on long‑term renal outcomes remain uncertain and warrant further study. These case reports documented hematological improvements after PEX, which may be attributed to its ability to clear pathogenic factors such as endothelial injury factors and autoantibodies and replace them with normal plasma components. This intervention could prevent severe thrombocytopenia and associated bleeding risks, improve the patient's overall clinical condition of patients, and facilitate definitive treatment options, including surgery [17,20]. In select cases, it may serve as a bridge, offering time for oncology intervention. We acknowledge the inherent limitations of a single case report, including the absence of long‑term follow‑up, incomplete renal recovery with persistent dialysis dependence, the unavailability of haptoglobin testing, the lack of quantitative schistocyte percentage on peripheral smear, and the inability to obtain detailed rectal cancer staging and pathology data from the external oncology hospital.

There are broader considerations of alternative management strategies for cancer‑related TMA. First, for patients with suspected complement dysregulation, eculizumab, a terminal complement inhibitor, has shown promising results in some cases of chemotherapy‑associated TMA. A systematic review of proteasome inhibitor‑induced TMA found that eculizumab significantly improved outcomes for patients unresponsive to initial PEX, achieving complete remission in seven cases [21]. Second, rituximab has been successfully used in refractory TTP and chemotherapy‑ associated TMA, including a case of mitomycin‑C‑induced TTP in a patient with rectal cancer who failed to respond to plasmapheresis and steroids [22]. Third, a retrospective study of 59 patients with cancer‑related TMA demonstrated that urgent chemotherapy was significantly associated with improved survival, while patients treated with eculizumab in addition to chemotherapy and PEX did not have longer overall survival or higher hematological remission rates [23]. We have incorporated these findings and emphasize that prompt anti‑cancer therapy remains the cornerstone of management, with PEX and other modalities playing a supportive or temporizing role only in selected diagnostic scenarios.

## Conclusions

This case highlights the diagnostic challenge of cancer-related TMA, which frequently involves the renal microvasculature, often leading to severe AKI. We provide a comprehensive medical process, from the initial clinical difficulty of not ruling out TTP to the crucial role of renal biopsy in confirming secondary TMA. Simultaneously, including the initiation of PEX treatment and tumor detection. This case provides valuable clinical insights into the diagnostic challenges and management considerations for cancer‑related TMA. First, it emphasizes that TMA-associated AKI can be the main manifestation of occult malignant tumors, which is an important consideration for nephrologists. Second, it provides a real-life example of differential diagnosis between TTP and other TMAs, leading to clinically significant differences in treatment. Finally, it contributes to the ongoing discussion on the limited role of PEX in cancer-related TMA. In our patient, PEX was associated with hematological improvement. However, because the patient remained dialysis‑dependent, the overall clinical benefit of PEX in this setting remains uncertain. The evidence supporting routine use of PEX in cancer‑related TMA is limited and controversial. Therefore, the role of PEX should be considered cautiously on a case‑by‑case basis, and treatment of the underlying malignancy remains the cornerstone of management. Our research findings aim to indicate that PEX can be considered in selected patients when TTP cannot be ruled out or when there is life-threatening thrombocytopenia present.
